# The Hologenome Concept of Evolution: Medical Implications

**DOI:** 10.5041/RMMJ.10359

**Published:** 2019-01-28

**Authors:** Eugene Rosenberg, Ilana Zilber-Rosenberg

**Affiliations:** Department of Molecular Microbiology and Biotechnology, Tel Aviv University, Ramat Aviv, Israel

**Keywords:** Beneficial microbes, genetic variation, holobiont, hologenome, microbiome

## Abstract

All natural animals and plants are holobionts, consisting of the host and microbiome, which is composed of abundant and diverse microorganisms. Health and disease of holobionts depend as much on interactions between host and microbiome and within the microbiome, as on interactions between organs and body parts of the host. Recent evidence indicates that a significant fraction of the microbiome is transferred by a variety of mechanisms from parent to offspring for many generations. Genetic variation in holobionts can occur in the microbiome as well as in the host genome, and it occurs more rapidly and by more mechanisms in genomes of microbiomes than in host genomes (e.g. via acquisition of novel microbes and horizontal gene transfer of microbial genes into host chromosomes). Evidence discussed in this review supports the concept that holobionts with their hologenomes can be considered levels of selection in evolution. Though changes in the microbiome can lead to evolution of the holobiont, it can also lead to dysbiosis and diseases (e.g. obesity, diarrhea, inflammatory bowel disease, and autism). In practice, the possibility of manipulating microbiomes offers the potential to prevent and cure diseases.

## INTRODUCTION

Biology is undergoing a paradigm change. Animals and plants can no longer be considered individuals.^1^ All are holobionts, consisting of the host and abundant and diverse microbiotas, which comprise bacteria, archaea, viruses, and protists. Symbioses between microorganisms and animals/plants, once thought to be rare events, are actually a hallmark of eukaryotic life.

A decade ago, we published the hologenome concept of evolution, which posits that the holobiont, in concert with its hologenome, is a level of selection in evolution.^2^,^3^ Considerable data have accumulated since the concept was first proposed that support and enrich it.^4^ Much of the data have come from the development of DNA-based techniques for analyzing bacterial communities, without having to culture them.

As soon as the right method was found, discoveries came as easily as ripe apples from a tree. (Robert Koch, 1905 Nobel Prize Laureate in Physiology or Medicine)

Though there have been a number of recent reviews on the role of the microbiome in human health and disease,^5^–^10^ in this review we wish to briefly describe the hologenome concept and discuss how it offers new perspectives to modern medicine.

Box A **Definitions**

***Bacterial species:*** the most widely used bacterial species definition is a group of strains showing over 97% of 16S rDNA gene-sequence identity.

***Holobiont:*** a term introduced by Margulis,^11^ which now refers to the host plus the entire microbiome.

***Hologenome:*** the union of all the genes in a holobiont, i.e. the host genome plus all of the genomes of the microbiome.^3^

***Horizontal gene transfer (HGT):*** transfer of groups of genes between bacteria of different taxa and from microbiomes to their hosts.

***Horizontal transmission of microbiota:*** transfer of microorganisms from parent to offspring via the environment.

***Microbiome:*** the community of symbiotic microorganisms.^12^

***Microbiota:*** microorganisms in the microbiome, but not necessarily all of it.

***Symbiosis:*** the term was first coined by Anton de Bary in the mid-nineteenth century as “the living together of different species.”^3^^(p.276)^ This broad definition is generally accepted, and easily comes to terms with the hologenome concept. The symbiotic system is usually constructed from a large partner termed the host and smaller partners called symbionts. A symbiont can be mutualistic, commensal, or pathogenic.

***Vertical transmission of microbiota:*** transfer from parent to offspring without mixing with microorganisms from the environment.^13^

## THE HOLOGENOME CONCEPT OF EVOLUTION

The hologenome concept of evolution is based on four generalizations:

1. All natural animals and plants are holobionts containing abundant and diverse microbiota.
2. Holobionts can function as distinct biological entities, anatomically, metabolically, immunologically, during development and in evolution. Microbiomes participate in achieving fitness of the holobionts.
3. A significant fraction of the microbiome genome together with the host genome is transmitted from one generation to the next, and thus can propagate unique properties of the holobiont.
4. Genetic variation in holobionts can occur by changes in the host and/or microbiome genomes. Since the microbiome genome can adjust to environmental dynamics more rapidly and by more processes than the host genome, it can play a fundamental role in adaptation and evolution of holobionts.

### Plants, Animals, and Humans are Holobionts

Development of non-culturing, DNA-based methods for analyzing bacterial communities has led to the determination of bacterial species diversity in a wide assortment of non-vertebrates, vertebrates, and plants. It is now clear that all natural animals and plants contain hundreds or thousands of different bacterial species as well as abundant viruses^14^ and often fungi^15^; the two last-mentioned have not been studied extensively to date. For example, it has been reported that the human gut contains 5,700 bacterial species,^16^ and human skin contains 1,000 species.^17^ These are minimum numbers since rare species cannot be determined by current methods. Because of the large diversity of bacterial species, the gut microbiome contains ca. 9 million unique protein-coding genes or 400 times more bacterial genes than human genes.^18^

One of the unexpected findings of studies of gut microbiomes is the enormous variation between individual humans; the bacterial species composition within the human gut is unique to each person. Nevertheless, microbiomes of different individuals are closer to each other than to microbiomes of other primates,^19^ suggesting that there is something common (a core) to the human microbiome. Shapira^20^ has discussed the differences between conserved core microbiota and flexible, environmentally driven microbiota, with regard to their maintenance and contributions to host adaptation. It should be emphasized that “presence” or “absence” of a bacterial species depends on technical limits of detection. Methods developed to detect rare species may reveal that there are many more common (core) species than currently considered and that individual variation may be the result of quantitative rather than qualitative differences that are caused by a different diet or other environmental factors. Foods, such as red wine,^21^ tea, coffee,^22^ and chocolate,^23^ food additives, such as food emulsifiers^24^ and artificial sweeteners,^25^ and essentially any material that is put in the mouth affect the gut microbiome at all ages. Also, microbiomes are affected by physical activity^26^ and illnesses, e.g. cancer^27^ and diabetes.^28^ Clearly, the complexity and dynamics of microbiomes are only beginning to be appreciated.

Microbiomes change also with age. Newborns are dominated by facultative anaerobes such as the Proteobacteria, after which the diversity of strict anaerobes within the Firmicutes and Bacteroidetes phyla increases towards a more adult-like profile by approximately one year.^29^,^30^ The microbiome in young children is shaped by mode of delivery,^31^ diet,^32^ exposure to environmental factors, such as furry pets,^33^ and, of course, antibiotic treatment.^34^ During most of adult life, the microbiome appears to be more or less stable.^35^ In older people (>65 years), however, the gut microbiome is extremely variable between individuals and differs from the microbiome of younger adults.^36^

### Microbiomes Affect the Fitness of Holobionts

A large number of studies have demonstrated the beneficial interactions between microbiomes and their hosts, leading to a better-adapted holobiont. Table 1 summarizes major contributions of the microbiome to holobionts.

**Table 1:** Examples of Microbial Participation in the Fitness of Holobionts.

Contribution of Microbiota	Examples
Protection against pathogens	Plants, animals, humans
Provides essential nutrients to host	SCFAs, AAs, vitamins
Fat storage and obesity	Shown in mice, chickens, and humans
Development	Squid light organ, legume nodule, immune system, angiogenesis, muscle thickness
Behavior	Brain, metabolites, hormones, stress, autism, sleep, mating selection, group living
Detoxification of toxic substances	Plant and fungal toxins in food, heavy metals

AA, amino acids; SCFAs, short-chain fatty acids.

A large number of studies have shown that resident microbes protect holobionts from pathogens. For example, following oral infection, the numbers of *Listeria monocytogenes* were 10,000-fold higher in the small intestine of germfree (GF) mice compared to conventional (CV) mice.^37^ Also *Staphylococcus aureus* infection is prevented by resident *Corynebacterium* species.^38^ Blocking binding sites and production of antibiotics are two common mechanisms by which resident bacteria protect the holobiont against pathogens.^39^,^40^ A strong argument for the role of bacteria in combatting infectious disease is the successful treatment of patients, suffering from severe diarrhea caused by *Clostridium difficile* infection, with fecal transplants from healthy donors (see further on).^41^

There are many examples of microbiomes contributing to their hosts by carrying out metabolic processes that the animal or plant is unable to carry out by itself. In humans, gut bacteria have been shown to perform many beneficial biochemical reactions, amongst them: (1) production of metabolites from dietary components, such as the conversion of dietary fiber to the short-chain fatty acids (SCFAs), acetate, propionate, and butyrate^42^ important for colonocyte health and regulatory activity of the body; (2) modification of metabolites that are produced by the host, such as converting primary bile acids to secondary bile acids, thus assisting in bile acid recycling^43^; (3) *de novo* synthesis of compounds, e.g. the important microbial immune modulator polysaccharide A, produced by the common gut bacterium *Bacteroides fragilis*^44^; and (4) synthesis of vitamin K as well as most of the water-soluble B vitamins.^45^

The human microbiotas play a role in energy metabolism and obesity, as will be discussed later in the review, and, although gut bacteria contributing to obesity are generally considered harmful, under certain conditions they are also beneficial. During the third trimester of pregnancy, these so-called “obese bacteria” become abundant^46^ and induce metabolic changes that promote energy storage in fat tissue that in turn encourages growth of the fetus and milk production in the mother. Also, during our evolution, food insecurity was a frequent occurrence, and the ability to store energy in the form of fat was probably advantageous for survival (“thrifty genotype hypothesis”^47^).

Symbiotic bacteria play a role in the development of animals and plants. For example, *Rhizobia* strains cooperate with legume plants to produce root nodules that perform nitrogen fixation,^48^ and *Vibrio fischeri* triggers the formation of the light organ in squid, where luminescence occurs helping the squid avoid predation. In humans and other vertebrates, the gut microbiome promotes the development of the immune system and body organs. Exposure to microorganisms educates the immune system, induces innate and adaptive immunity,^49^ and initiates memory B and T cells that are essential to combat various pathogens. In addition, the gut microbiome encourages the development of bone mass^50^ and blood vessels in the intestinal wall.^51^

Gut microbiotas modulate brain development and behavior, including anxiety and mood disorders,^52^,^53^ as will be discussed later in the review. Microbial gut–brain signaling is bidirectional. The circuitry of neurons, hormones, and chemical neurotransmitters enables messages to be transmitted between the brain and the gut. The gut microbiota influences the body’s level of the potent neurotransmitter serotonin, which promotes feelings of happiness and peacefulness.^54^ Conversely, the rate at which food is being moved and how much mucus is lining the gut—both of which can be controlled by the brain—have a direct impact on gut microbiota.

### Transmission

The mechanism for transmission of host DNA to offspring is well understood and need not be discussed here. Transmission of the microbiome from parent to offspring also occurs, but with a variety of mechanisms: (1) vegetative reproduction in plants and many animals, such as worms and corals (vertical transmission); (2) via oocytes in sponges, herbs, and many insects (vertical transmission); (3) coprophagy (eating the feces of parents) in many animal species (both vertical and horizontal transmission); (4) physical contact starting at birth in most animals, including humans (both vertical and horizontal); and (5) mother’s milk in mammals (vertical).

In humans, transmission to the offspring occurs initially via inoculation with maternal vaginal and fecal microbes when the baby transits the birth channel (vertical transmission). Kissing and hugging provides additional microbiota to the offspring. Breastfeeding is an additional route of maternal vertical microbial transmission.^55^ Human milk contains ca. 10^5^ bacteria per mL, composed of hundreds of species.^56^ Analyses of the DNA of several bacterial strains isolated from mother’s milk demonstrated that they were identical to those found in the offspring,^57^ providing support for vertical transmission. Mother’s milk is also a continuous source of modified oligosaccharides that support the growth of these bacteria but are not digestible by the infant.^58^ In essence, these oligosaccharides function as natural prebiotics. The *Bifidobacterium* species contain unique genetic loci responsible for vigorous growth on these oligosaccharides,^59^ suggesting a remarkable co-evolution between the symbiotic bacteria and their human host, benefiting both.

Long-term transmission of microbiota in great apes, including humans, was studied using both 16S ribosomal gene sequences^60^ and rapidly evolving gyrB gene sequences.^61^ The host species phylogenies based on the composition of these microbial communities were completely congruent with the known evolutionary relationships of the hosts. The authors concluded that over evolutionary timescales the composition of the gut microbiota among great ape species is phylogenetically conserved and has diverged in a manner consistent with vertical inheritance.

### Genetic Variation and Evolution of Holobionts

Genetic variation occurs more rapidly in genomes of microorganisms than in host genomes, thereby offering a potential for manipulating the microbiome to prevent and treat certain diseases of holobionts. Genetic variation in holobionts can occur, in addition to mutation and DNA rearrangement, also by three other mechanisms: (1) amplification or reduction of specific microbes, (2) acquisition of novel microbes from the environment, and (3) horizontal gene transfer from microbes to microbe or from microbe to host.

Amplification/reduction refers to the increase/ decrease of one group of symbionts relative to others. This can occur rapidly when conditions change. An increase in the number of a particular microbe is equivalent to amplification of a whole set of genes. Considering the large amount of genetic information encoded in the diverse microbial population of holobionts, microbial amplification/reduction can be a powerful mechanism for contributing to adaptation, development, and evolution of holobionts. Since genetic variation by amplification is driven by the environment, it has a Lamarckian aspect to it.^62^ Amplification is also a crucial step in genetic variation by acquisition of novel microbes because pioneer microbes need to amplify in order to become established in its host.

Animals, including humans, and plants come into contact with billions of microorganisms during their lifetime, via air, water, and interaction with organic and inorganic surfaces. Occasionally some of these microbes will find a niche and under appropriate conditions will amplify in the host and become part of the microbiome. Acquisition of a microbe introduces hundreds of new genes into the holobiont. Rather than trying to create genes that have already evolved in microbes, animals and plants acquired pre-evolved genetic information in the form of microbes. Microbes were on this planet for 2.1 billion years before there were any animals or plants. During this time, they developed into organisms encompassing enormous biochemical diversity. The first eukaryote was probably formed by the acquisition of bacteria to eventually form mitochondria^63^ and chloroplasts.^64^ Uptake of microbes into multicellular organisms continued to provide genetic variation for holobionts throughout evolution.

An example of a major evolutionary event that was driven by the acquisition of bacteria is the ability of many animals, including humans, to use plant fibers as nutrients.^65^ However, animal genomes do not contain the information for synthesizing enzymes that degrade the complex polysaccharides in plant material. Instead, they rely on microorganisms that are present in their digestive tract. These microbes anaerobically convert polysaccharides to fatty acids that are a source of carbon and energy for their host animal. It is likely that these bacteria were acquired by a gradual process of internalizing them from the soil. Instead of plant fiber being broken down in the soil prior to ingestion, it “rots” in the gut after consumption.

Another important mode of genetic variation in holobionts, referred to as horizontal gene transfer (HGT), involves the transfer of groups of genes between bacteria of different taxa and from microbiomes to their host. Intimate contact between microbes and between microbes and host in holobionts would promote HGT. It has been suggested that nutritional adaptation is one of the key selective pressures on the microbiome in the mammalian gut, and that HGT processes contribute to that adaptation.^66^ Until 2010, only a few examples of HGT events were recognized in which genes from microbes were transferred sometime in the past to animals. These included transfer of: *Wolbachia* genes to the chromosomes of their insect hosts,^67^ bacterial and fungal genes into the telomere region of rotifers,^68^ fungal genes to aphids,^69^ and cellulose genes from bacteria to nematodes.^70^ However, an examination of a large number of high-quality genomes, that became available recently, has led to the conclusion that HGT in animals and plants was a general phenomenon that resulted in the incorporation of tens or even hundreds of active foreign genes into the eukaryotic genome.^71^

In humans, 145 genes (not present in other primates) were attributed to HGT.^72^ These genes are distributed throughout the genome and play a variety of roles, such as amino-acid metabolism (2 genes), macromolecule modifications (15 genes), lipid metabolism (13 genes), antioxidant activities (5 genes), and innate immune response (7 genes). Most of the 145 genes identified in the study came from bacteria, but some originated from viruses and yeasts. Analysis of a moss identified 128 genes found in land plants but absent from algae.^73^ These genes were acquired by HGT from prokaryotes, fungi, or viruses. Many of these genes are involved in some essential plant-specific activities, such as xylem formation, plant defense, nitrogen recycling, and the biosynthesis of starch, polyamines, hormones, and glutathione.

A key event in the evolution of placental mammals was the acquisition by HGT, from a retrovirus, of the gene coding for the protein syncytin.^74^ Initially, the function of syncytin was to allow retroviruses to fuse host cells so that viruses could move from one cell to another. Now, syncytin is necessary for the development of the placental syncytium, an essential part of the mother–fetus barrier. In addition, retroviral-derived DNA appears to have played a crucial function in the generation of the progesterone-sensitive uterine decidual cell, allowing nutrient provision to the developing embryo.^75^ These data indicate that the integration of viral DNA into host genomes by HGT played a primary role in major evolutionary events.

Horizontal gene transfer (HGT) from microbe to microbe can also affect human metabolism and evolution. An example is the ability of Japanese to break down agar (an abundant ingredient in their diet) since they have a bacterium in their gut that contains genes that code for the porphyranases that degrade the polysaccharide agarose of agar. Westerners lack this bacterium in their gut and therefore cannot digest agar. The group of genes coding for agarose digestion was driven into a resident gut bacterium by HGT from a marine bacterium that was present on raw seaweed.^76^ Bacteria with the transferred genes spread throughout the Japanese population by vertical and horizontal transmission.

In summary, the evidence brought forth up to now in this review supports the concept that holobionts with their hologenomes can be considered levels of selection in evolution. Furthermore, genetic variation and the evolution of holobionts involve, in addition to classical genetic variations in the host, also acquisition of novel microbes and HGT of microbial genes into host chromosomes. In the following section, we will be discussing the relationship between the hologenome concept and some human diseases, thereby reinforcing the holobiont as a unified biological entity.

## HOW DOES THE HOLOGENOME CONCEPT RELATE TO MEDICINE?

Although we are only beginning to understand what constitutes a healthy microbiome, there is already considerable data indicating significant differences between microbiomes of diseased compared to healthy holobionts. Pitlik and Koren^77^ have hypothesized that probably every illness of holobionts, acute or chronic, is accompanied by some perturbation of the microbiome. This hypothesis is a logical outcome of the hologenome concept and is based on the tight interdependence of the host and its microbiome. Any disturbance in the delicate equilibrium of host–microbiome interactions can on the one hand participate in disease causation, or, on the other hand, because of changing internal conditions, be a result of it.

In previous sections of this review, we have described beneficial aspects of the microbiomes, those that participate in the functioning of healthy holobionts, as we know them. In this section, we wish to discuss the evidence demonstrating that alteration of the microbiome can lead to dysbiosis and disease of the holobiont, and, in selected cases, exemplifying general principles, we will examine how they do it. We will also discuss how manipulation of the microbiome has the potential to prevent or cure certain diseases by reaching a new healthier equilibrium.

### Obesity and Metabolic Diseases

The twin epidemics of obesity and type 2 diabetes have generated considerable research and information in the last 20 years, including a growing awareness of the role of the microbiome. Understanding of the complex metabolic interplay observed within the holobiont led to milestone studies, more than a decade ago, showing the involvement of microbiota in fat storage^78^ and the existence of a different microbiota in obese humans as compared to normal-weight people.^79^ These studies also demonstrated reduced microbial diversity in obesity, altered bacterial genes and pathways,^80^ and an abundance of Firmicutes compared to Bacteroidetes.^81^ The picture perceived today, regarding the involvement of the microbiome in obesity of humans, seems to be more complex and depends on several variables, such as on the technique used for determining bacterial species and numbers, the age, sex, and ethnicity of the subjects, and the location of the microbes in the gut.^82^,^83^ It may well be that differences in microbiotas also depend on extent of obesity, becoming more pronounced as the BMI is elevated and abdominal fat is greater, as has been shown comparing microbial gene richness in people with very high BMI and those with lower BMI.^84^ Because of the complex interactions of gut microbiota with the human body, the mechanisms underlying the network between obesity and microbiota probably are manifold and heterogeneous. The most likely mechanisms were summarized in a number of reviews^85^–^87^ and include: increase in energy harvest by the “obese microbiome”; lower short-chain fatty acid production influencing a cascade of regulatory activities; greater intestinal permeability causing leakage of bacterial endotoxins into the blood; low-grade inflammation affecting adipogenesis and other targets; impact on toll-like receptors; and increased endocannabinoid receptor system tone.

Obese microbiotas with their metabolic effects have been implicated in metabolic diseases especially in the metabolic syndrome and in type 2 diabetes, in addition to cardiovascular disease and fatty liver.^5^,^87^–^90^ Obese microbiotas have also been implicated in certain types of cancer.^91^,^92^ It should be noted that in spite of their pathological role, “obese bacteria” are also associated with weight gain during the third trimester of pregnancy, as noted in a previous section, so caution should be used in considering these obese bacteria as only being harmful.^46^

The empirical demonstration that microbiotas can have a causal effect on obesity and other metabolic diseases has inspired investigators to test probiotics and prebiotics for the treatment of these diseases. Most of the published experiments on obesity treatment were with animals. The few human studies have been inconclusive.^87^,^93^ The potential of microbiome-targeted therapies for obesity and metabolic diseases has recently been reviewed,^87^,^94^ and therapies include probiotics, prebiotics, and fecal transplant, in addition to indirect effects such as via medications or metabolic surgery. In our opinion, based on the hologenome concept of genetic variation by amplification/reduction, successful prevention or treatment of obesity and metabolic diseases by changing microbiota will require a parallel change in gut environment. For instance, maintaining an appropriate long-term diet, thereby conserving a useful concentration of the beneficial microbiota.

### Diarrhea Caused by Clostridium difficile

The major success in the use of microbiota to treat disease is fecal transplants from healthy donors for the treatment of diarrhea caused by *Clostridium difficile*. Fecal microbiota transplantation (FMT) is a treatment that involves administration of minimally manipulated microbial community from stool of a healthy donor into the patient’s intestinal tract. The transplant material can be delivered by various routes, including an encapsulated form as an oral medication.^95^ Hundreds of patients with reoccurring diarrhea caused by antibiotic-resistant *C. difficile* have been treated by FMT with an average cure rate of 90.4%.^41^ A recent study on a small number of patients reinforced previous findings that fecal transplantation was also better than antibiotics for primary treatment of *C. difficile* infections.^96^

Most of the patients that were treated by FMT had previously been exposed to multiple courses of antibiotics. Not surprisingly, these patients had a greatly disturbed microbiome, with a complete disappearance of Bacteroidetes, reduction in Firmicutes, increase in Proteobacteria, and a general loss of microbial diversity.^97^ Fecal microbiota transplantation (FMT) normalizes the composition and functionality of gut microbiota as early as 24 h after treatment.^98^,^99^ Four mechanisms have been suggested to explain how FMT restores normal gut microbial community structure and functionality:^100^ (1) Direct competition of healthy commensal microbiota delivered by FMT with *C. difficile* for space and nutrients; (2) restoration of secondary bile acid metabolism in the colon; (3) repair of the gut barrier by stimulation of the mucosal immune system; and (4) bacterial production of bacteriocins, such as thuricin and nisin, which are antimicrobial peptides with bactericidal activity against *C. difficile*.^101^,^102^

### Inflammatory Bowel Disease (IBD)

Recent studies using improved DNA technology have shown that IBD is associated with characteristic shifts in the composition of the gut microbiome, suggesting that IBD results from altered interactions between intestinal microbes and the mucosal immune system.^103^ The concept of an altered collective gut microbiota rather than identification of a single culprit is possibly the most significant development in IBD research. In general, IBD studies show an overall decrease in biodiversity, a decreased representation of several taxa within the Firmicutes phylum, and an increase in the Gammaproteobacteria, which were also found in pairs of monozygotic twins discordant for Crohn’s disease (CD).^104^ There is a reduced diversity in inflamed versus non-inflamed areas of the gut, even within the same patient, and CD patients have a lower overall bacterial abundance at inflamed regions.^105^

Studies on how manipulation of the microbiome can be used to treat IBD fall into three broad categories: (1) antibiotics to reduce inflammatory bacteria, (2) modification of the gut environment through diet and prebiotics to encourage the growth of beneficial bacteria, and (3) direct introduction of beneficial bacteria via probiotics or FMT. Epidemiological, experimental, and clinical evidence of the current status of these three potential treatments has recently been reviewed, leading to the conclusion that “it is currently challenging to elegantly translate results into clinical practice.”^106^,^107^^(p.26)^

### Autism Spectrum Disorders (ASDs)

Autism spectrum disorders (ASDs) are neurodevelopmental conditions characterized by social and behavioral impairments, often accompanied by gastrointestinal abnormalities. Several studies have shown an altered gut microbiome associated with ASDs, both in bacterial and fungal communities.^108^–^110^ Autistic children have a significant increase in the Firmicutes/Bacteroidetes ratio due to significant reduction of Bacteroidetes. At the genus level, autistic subjects have a significantly higher abundance of *Collinsella*, *Corynebacterium*, *Dorea*, *Lactobacillus*, and *Candida*, and a reduced abundance of *Alistipes*, *Bilophila*, *Dialister*, *Parabacteroides*, and *Veillonella* compared to healthy subjects.

Evidence suggests an early immune activation with chronic inflammation and cytokine dysregulation in ASDs.^111^ In animal studies, it has been shown that systemic inflammation induced by lipopolysaccharide provokes behavioral changes and impairs the blood–brain barrier.^112^ One of the strongest arguments for the role of bacteria in ASDs is the report that oral treatment of mice that display features of ASD with the human commensal *Bacteroides fragilis* corrects gut permeability, alters microbial composition, and ameliorates defects in communicative, anxiety-like, sensorimotor, and other stereotypic behaviors.^113^ Furthermore, the treatment altered several metabolites that are known to affect the brain. These data support a general gut–microbiome–brain connection which is pathologically pronounced in ASD, and suggest potential probiotic therapy for human ASDs, and possibly other behavioral problems.

## CONCLUSIONS

There is now abundant evidence supporting the hologenome concept, which posits that the holobiont with its hologenome is a level of selection in evolution. However, the complexity and dynamics of holobionts are only beginning to be understood. What is clear is that most environmental elements affecting the holobiont, such as materials entering the digestive tract, can alter the microbiome and lead to adaptation and evolution, but also to dysbiosis and disease. Many diseases can only be understood by considering the patient as a holobiont, a complex system of interacting microbes and host. Moreover, the microbiome interacts with the host as do organs and body parts. Most of the time the interaction benefits the host, but sometimes it also causes harm. In practice, one of the challenges of modern medicine is to manipulate the microbiome so that it will prevent or cure certain diseases.

## Abbreviations

ASDs: autism spectrum disorders
CD: Crohn’s disease
IBD: inflammatory bowel disease
FMT: fecal microbiota transplantation.
